# Case report of MR perfusion imaging in Sinking Skin Flap Syndrome: growing evidence for hemodynamic impairment

**DOI:** 10.1186/1471-2377-10-80

**Published:** 2010-09-11

**Authors:** Andre Kemmling, Thomas Duning, Lars Lemcke, Thomas Niederstadt, Jens Minnerup, Heike Wersching, Martin Marziniak

**Affiliations:** 1 Institute for Clinical Radiology, University Hospital of Muenster, Albert-Schweitzer-Strasse 33, 48149 Muenster, Germany; 2 Department of Neurology, University Hospital of Muenster, Albert-Schweitzer-Strasse 33, 48149 Muenster, Germany; 3 Department of Neurosurgery, University Hospital of Muenster, Albert-Schweitzer-Strasse 33, 48149 Muenster, Germany

## Abstract

**Background:**

The syndrome of the sinking skin flap (SSSF) with delayed sensorimotor deficits after craniectomy is not well known and often neglected. Among various postulated causes, there is evidence that disturbed brain perfusion may be related to the observed symptoms, and that cranioplasty reliably alleviates these symptoms. We report a case of sinking skin flap syndrome (SSFS) with recovery from neurological sensorimotor deficits after cranioplasty correlated with pre- and postsurgical MR brain perfusion studies.

**Case Presentation:**

A 42-year-old woman presented with slowly progressive sensorimotor paresis of her left arm after decompressive extensive craniectomy due to subarachnoid hemorrhage four months ago. Her right cranium showed a "sinking skin flap". After cranioplastic repair of her skull defect, the patient fully recovered from her symptoms. Before cranioplasty, reduced brain perfusion in the right central cortical region was observed in MR-perfusion images. After cranioplasty, a marked increase in brain perfusion was observed which correlated with objective clinical recovery.

**Conclusion:**

There is increasing evidence that impaired blood flow is responsible for delayed motor deficits in patients with sinking skin flap syndrome in the area of compressed brain regions. Symptoms should be evaluated by brain perfusion imaging complementing surgical decision-making.

## Background

The "sinking skin flap syndrome" (SSFS) is characterized by neurological symptoms (headache, epileptic seizures, vertigo, dysesthesias, or paresis) following extensive decompressive craniectomy which improve after cranioplasty. There are few reports of SSFS associated with delayed motor deficits, designated as "motor trephine syndrome",. The "sinking skin" is a result of lower intracranial pressure in upright position and higher atmospheric pressure so that the pressure gradient across the skin flap directly acts on the brain resulting in tissue compression. Accordingly, neurological improvement has been reported when this pressure disturbance is normalized temporarily with change in posture[1,2] or permanently after cranioplastic repair[3-6]. There are various pathophysiological theories for the cause of neurological deficits[7]: *a) *direct cortical compression[8,9]; *b*) hydrodynamically disturbed cerebrospinal fluid parameters (CSF pressures and elastance) linked to changes in posture[4,7,10]; *c) *hemodynamically reduced cerebral blood flow, cerebrovascular reserve capacity, and venous return as a result of pressure on the vasculature and brain tissue[6,11-13]; *d) *disturbed metabolism[14,15].

Radiological studies and case series correlating brain perfusion with neurological deficits before and after cranioplastic repair are limited[6,11-13,15-18]. Nonetheless, all reports demonstrate significant improvement of brain perfusion concurrent with neurological recovery after cranioplasty supporting the theory of impaired hemodynamics.

A case of motor trephine syndrome assessed by MR perfusion (MRP) study before and after cranioplasty is presented.

## Case Presentation

A 42-year-old woman presented with slowly progressive weakness and dysesthesia of her left arm after decompressive craniectomy. Neurological examination revealed a slight, distal paresis with increased tendon reflexes, dysesthesia of her left arm, and reduced fine motor skills of the left hand. She suffered from a subarachnoid hemorrhage (SAH) due to a spontaneously ruptured aneurysm of the right middle cerebral artery 4 months ago. A right fronto-temporo-parietal craniectomy with evacuation of the hematoma and coiling of the aneurysm had been performed as initial treatment, and the bone flap had not been replaced. Initial postsurgical left hemiparesis completely disappeared, but late sensorimotor disturbances of the left arm continuously worsened over the last three weeks. The skin of the right temporoparietal region was markedly sunken due to the large skull defect.

Computed tomography and magnetic resonance imaging of the brain were unremarkable except for minor focal gliosis. There were no signs of ischemia or edema, new hemorrhage, coil extrusion, brain shift, or disturbed CSF circulation. Three consecutive electroencephalograms did not show epileptiform discharges, an extra- and transcranial Doppler ultrasonography was unrevealing.

A secondary neurological deterioration was diagnosed due to SSFS.

After replacement of the bone flap the sensorimotor deficits of her left arm continuously improved within a period of 10 to 14 days. At the follow-up examination four weeks after cranioplasty, the patient reported a complete recovery. Only slightly increased tendon reflexes of the left arm were still present.

Brain perfusion immediately before and four weeks after cranioplastic repair was assessed by T2*-weighted MR-perfusion imaging (Philips Intera 1.5-T MR system, TR 17, TE 8, flip angle 7, EPI factor 17, FOV 220 mm, 22 slices, slice thickness 6, matrix 128) with a double dose of Gadovist-1.0 (0.2 ml/kg body weight, 5 ml/s). Perfusion maps were calculated on a Philips ViewForum workstation from regions of interest (ROI) drawn over the entire left and right hemisphere for each slice, respectively. For objective comparison of perfusion parameters between the two consecutive time points, relative cerebral blood flow (CBF), cerebral blood volume (CBV), and mean transit time (MTT) of the right hemisphere compared to normal left side were calculated.

**Figure 1:**
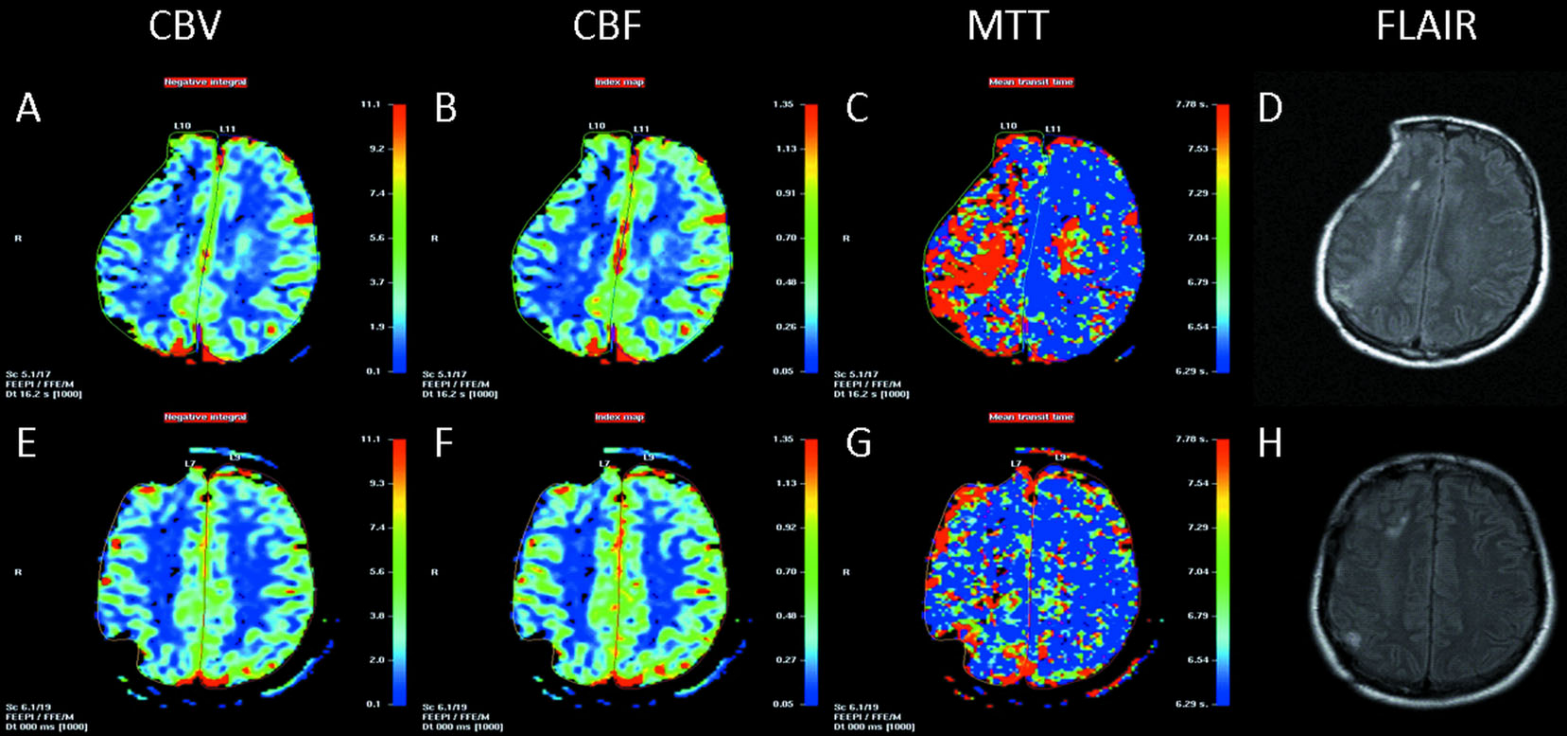
**MR-perfusion maps and anatomy before (A-D) and after (E-F) cranioplastic repair of a patient with the "sinking skin flap syndrome" with delayed motor deficits**. A focal area of hypoperfusion (reduced blood flow and blood volume, and increased mean transit time) is observed in the right parietal cortex (A-C) beneath the skin flap. After cranioplasty, brain perfusion maps show no longer show a significant perfusion deficit.

**Figure 2:**
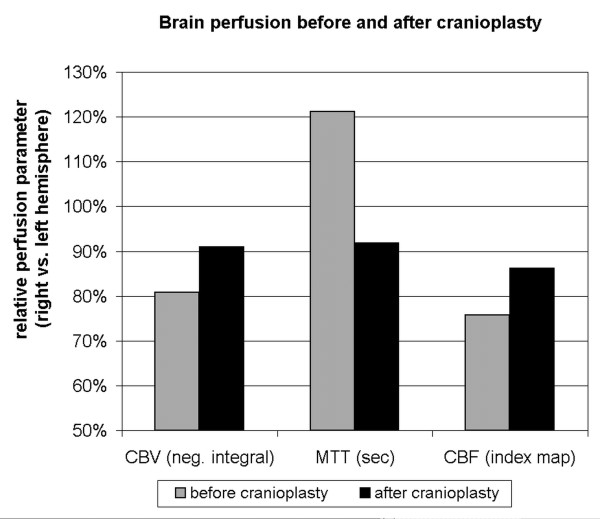
**Brain perfusion in the right hemisphere compared to healthy left side before and after cranioplastic repair in a patient with "sinking skin flap syndrome"**. Improvement of all three perfusion parameters was observed (relative cerebral blood volume and blood flow increased, relative mean transit time decreased).

Before cranioplasty brain perfusion in the right hemisphere was lower compared to the left side. Perfusion maps demonstrated a focal deficit particularly in the compressed central cortical region beneath the skinflap (Fig 1). Right hemispheric perfusion improved after cranioplastic repair (Fig 2), relative blood flow and blood volume increased and mean transit time decreased (rCBF 0.76 vs. 0.86; rCBV 0.81 vs. 0.91; rMTT 1.21 vs. 0.92; before vs. after cranioplasty, respectively).

## Discussion

Sinking skin flap syndrome with delayed motor deficits, or "motor trephine syndrome" is not well known in patients with large skull defects, where progressive neurological deterioration is associated with the sinking skin flap[4,12]. In the present case, sensorimotor paresis promptly reversed after cranioplastic repair and MR brain perfusion imaging correlated well with objective clinical assessment. Improvement of brain perfusion concurrent with neurological recovery supports the theory of impaired hemodynamics as the underlying pathomechanism. SSFS should be considered in the differential diagnosis of secondary neurological deterioration after decompressive craniectomy.

Evaluation of SSFS with brain perfusion is not commonly performed; only few cases with perfusion imaging before and after cranioplasty have been reported correlating clinical symptoms with hemodynamics in the affected hemisphere. Winkler et al. demonstrated improved blood flow and cerebrovascular reserve capacity after cranioplasty in 12 cases using transcranial Doppler sonography and 18-FDG positron emission tomography[14]. In small case series reporting neurological deficits after early decompression from traumatic brain injury or subarachnoid hemorrhage, serial xenon-CT studies showed continuous improvement of CBF concurrent with neurological recovery after cranioplasty[11,18]. Likewise, in seven cases after cranioplastic repair, Yoshida et al. could show a link between improved brain perfusion and cerebral metabolism using xenon-CT and P31 magnetic resonance spectroscopy[15]. In a series of six patients, higher peak value and shorter washout in dynamic contrast enhanced CT scans before and after cranioplasty were correlated with improved aphasia, motor function, and level of consciousness[6]. A case of sunken skin flap related to unilateral spatial neglect was assessed by SPECT showing concordant improvement of CBF and cognitive deficit after cranioplasty[17]. Two cases of motor trephine syndrome had rapid improvement of contralateral hand function within five days after cranioplastic repair, and this was coincident with improved CBF, CBV, and MTT in first-pass quantitative CT perfusion imaging[12]. So far, this is the first report using MR-perfusion for assessment of delayed motor deficits after craniectomy.

Even though perfusion imaging generally correlates well with symptoms in SSFS, it must be noted that an inherent limitation may be a result of extensive parenchymal damage or gliosis leading to unspecific perfusion asymmetry.

The only systematic prospective study of SSFS revealed an incidence of 30% of SSFS[8] whereas a second retrospective series reported an incidence of 7.8% in 64 craniectomized patients[19]. Therefore, it may be hypothesized that because of lacking knowledge SSFS is often over-looked. One reason might be the delayed onset of symptoms after the patient has already left the rehabilitation units, another reason might be that SSFS is not very commonly known.

Furthermore, it is to be expected that decompressive craniectomies will be carried out more often in the future due to the improvement of functional outcome after malignant middle cerebral artery infarction[20] and SSFS will become increasingly important. In the presented case, cranioplasty after 4 months lead to a prompt neurological recovery from delayed sensorimotor deficit without postsurgical complications. This result emphasizes the potential benefit of even earlier cranioplasty which should be considered particularly in patients with SSFS and delayed motor deficit.

## Conclusion

This case report contributes to emerging evidence that impaired brain perfusion is a significant mechanism in the sinking skin flap syndrome after decompressive craniectomy. Cranioplasty improves brain perfusion alongside with neurological deficit. Our results emphasize that using brain perfusion imaging could complement surgical decision making for cranioplastic repair.

## Consent

Written informed consent was obtained from the patient for publication of this case report and any accompanying images. A copy of the written consent is available for review by the Editor-in-Chief of this journal.

## Competing interests

The authors declare that they have no competing interests.

## Authors' contributions

AK, TD: have made substantial contributions to conception and design, acquisition, analysis and interpretation of data; have been involved in drafting the manuscript or revising it critically for important intellectual content. LL, TN: have made substantial contributions to acquisition, analysis and interpretation of clinical and imaging data. HW, JM: have been involved in drafting the manuscript or revising it critically for important intellectual content. MM: has given final approval of the version to be published; has made substantial contributions to acquisition, analysis and interpretation of clinical data. All authors have read and approved the final manuscript.

## Pre-publication history

The pre-publication history for this paper can be accessed here:

http://www.biomedcentral.com/1471-2377/10/80/prepub
